# Activity of a novel antimicrobial peptide against *Pseudomonas aeruginosa* biofilms

**DOI:** 10.1038/s41598-018-33016-7

**Published:** 2018-10-03

**Authors:** Trevor Beaudoin, Tracy A. Stone, Miroslawa Glibowicka, Christina Adams, Yvonne Yau, Saumel Ahmadi, Christine E. Bear, Hartmut Grasemann, Valerie Waters, Charles M. Deber

**Affiliations:** 1 Division of Translational Medicine, Research Institute, Hospital for Sick Children, Toronto, Canada; 2 Division of Molecular Medicine, Research Institute, Hospital for Sick Children, Toronto, Canada; 3 Department of Biochemistry, University of Toronto, Toronto, Ontario, Canada; 4 Department of Physiology, University of Toronto, Toronto, Ontario, Canada; 5 Division of Microbiology, Department of Pediatric Laboratory Medicine, Hospital for Sick Children, Toronto, Canada; 6 Department of Laboratory Medicine and Pathobiology, University of Toronto, Toronto, Ontario, Canada; 7 Division of Respiratory Medicine, Department of Pediatrics, Hospital for Sick Children, Toronto, Canada; 8 Division of Infectious Diseases, Department of Pediatrics, The Hospital for Sick Children, University of Toronto, 555 University Avenue, Toronto, M5G 1X8 Canada

## Abstract

With the increasing recognition of biofilms in human disease, the development of novel antimicrobial therapies is of critical importance. For example, in patients with cystic fibrosis (CF), the acquisition of host-adapted, chronic *Pseudomonas aeruginosa* infection is associated with a decline in lung function and increased mortality. Our objective was to test the *in vitro* efficacy of a membrane-active antimicrobial peptide we designed, termed 6K-F17 (sequence: KKKKKK-AAFAAWAAFAA-NH_2_), against multidrug resistant *P*. *aeruginosa* biofilms. This peptide displays high antimicrobial activity against a range of pathogenic bacteria, yet is non-hemolytic to human erythrocytes and non-toxic to human bronchial epithelial cells. In the present work, *P*. *aeruginosa* strain PAO1, and four multidrug resistant (MDR) isolates from chronically infected CF individuals, were grown as 48-hour biofilms in a static biofilm slide chamber model. These biofilms were then exposed to varying concentrations of 6K-F17 alone, or in the presence of tobramycin, prior to confocal imaging. Biofilm biovolume and viability were assessed. 6K-F17 was able to kill biofilms – even in the presence of sputum – and greatly reduce biofilm biovolume in PAO1 and MDR isolates. Strikingly, when used in conjunction with tobramycin, low doses of 6K-F17 significantly potentiated tobramycin killing, leading to biofilm destruction.

## Introduction

Cystic fibrosis (CF) is a genetic disease arising from mutations in the cystic fibrosis transmembrane conductance regulator (CFTR) gene that encodes a chloride ion transporter^1^. Impaired trans-epithelial chloride transport leads to dehydrated airway secretions and a lack of airway mucus clearance^2,3^. As a result, individuals with CF are prone to repeated bacterial infections by different pathogens, leading to bronchiectasis and respiratory failure^3,4^. In particular, chronic infection with *Pseudomonas aeruginosa* has been shown to lead to more rapid lung function decline and premature death^5,6^. Once *P*. *aeruginosa* establishes chronic infection, it undergoes a number of adaptations, including the formation of biofilms and the expression of multidrug resistance pumps, making infections difficult to treat with standard antibiotic therapy^7–14^.

Progress toward developing treatments for biofilm infections has been made through the use of cationic antimicrobial peptides (CAPs), which are found naturally in a wide variety of organisms and constitute a major component of the innate immune system^15–17^. There are a number of possible mechanisms of action of antimicrobial peptides in inhibiting or eradicating bacterial biofilms. The anti-biofilm activity of antimicrobial peptides may be the result of a classic microbicidal effect (usually occurring at concentrations equal or higher than minimum inhibitory concentration (MIC)) as well as through non-classical mechanisms of action (at concentrations much lower than MIC)^18^. The microbicidal effect is due primarily to the permeabilization of the bacterial cell membrane of either detached planktonic or biofilm embedded bacteria^19–21^. Antimicrobial peptides can also target the biofilm mode of growth by inhibiting bacterial adhesion (to surfaces or other bacteria)^22,23^, interfering with gene expression (including genes related to motility, matrix synthesis and quorum sensing)^24,25^ and modulating the host response (such as the activity of host immune cells and the release of inflammatory cytokines)^26^.

Given their mechanism of action, CAPs may effectively target biofilms, as their activity is independent of growth rate, metabolic activity or the presence of persister cells^27,28^. Naturally occurring peptides have adapted to perform in a specific environmental niche, and thus are limited in their ability to function in settings such as the CF lung^15–17^. In contrast, synthetically engineered CAPs can often be developed to overcome these limitations^29^. In the present study we report the ability of 6K-F17 – a peptide we designed from first principles of hydrophobicity, net positive charge, and sequence patterning^30,31^ – to effectively kill mature biofilms of *P*. *aeruginosa* from chronically infected CF patients, grown in static culture with and without CF sputum, along with the power of the peptide to potentiate the bactericidal activity of tobramycin, a commonly used inhaled antibiotic in CF *P*. *aeruginosa* infections^32^.

## Results

### Properties of the antimicrobial peptide 6K-F17: Safety toward mammalian cells

Essentially all natural CAPs consist of cationic amphipathic sequences with Lys (and/or Arg) residues distributed throughout the sequence, which when folded into an α-helix upon membrane association, presents a lipid-interactive hydrophobic face^33^. In contrast, the synthetic CAP contains positive (Lys) residues clustered at the peptide N-terminus, with the remainder of the sequence consisting of an uninterrupted hydrophobic segment (6K-F17; sequence KKKKKK-AAFAAWAAFAA-NH_2_) which is designed to maximize insertion into, and cause physical disruption of, bacterial membranes^34,35^. The membrane-penetrating power of this peptide likely derives from this implementation of charge segregation from the hydrophobic core^30^. As such, the designed CAPs operate by a non-specific bacterial (but not mammalian) membrane disruption mechanism that is unlikely to evoke rapid resistance, suggesting that CAPs of the present design can prolong their therapeutic time frame.

Yet 6K-F17 is remarkably selective for bacterial rather than host membranes and, in contrast to most natural amphipathic CAPs, is non-hemolytic to human erythrocytes up to concentrations >500 μg/ml^31^. This selectivity devolves from the fact that once attracted electrostatically to the anionic surface of a bacterial membrane, the effective hydrophobicity of 6K-F17 is above the threshold for spontaneous membrane penetration, while in the absence of electrostatic attraction, this level of hydrophobicity *per se* is below the threshold for penetration of the zwitterionic membranes of mammalian cells^30^. Because the 6K-F17 peptide consists of the natural L-isomers of common amino acids it can be efficiently synthesized and is rendered water-soluble by the six polar Lys residues, thereby facilitating characterization and purification.

Examples of 6K-F17 MICs against the *P*. *aeruginosa* lab strain PAO1, and against several clinical *P*. *aeruginosa* strains as studied herein (*vide infra*), are presented in Table 1, and compared to corresponding MICs for the conventional antibiotic tobramycin; in several instances, the 6K-F17 MICs are equal or less than the tobramycin values.

**Table 1:** Minimum inhibitory concentration of 6K-F17 and tobramycin against *P*. *aeruginosa* isolates^a^.

Isolate	6K-F17 (µg/mL)	Tobramycin (μg/mL)
PAO1	2	0.64
005E3-2	64	>128
007E3-2	8	8
014B2-1	256	>128
035B7-1	16	>128

^a^Experimental procedures are as previously described for MICs for 6K-F17 against lab strain PAO1^30,31^. See Methods section for details.

The activity of CAPs may be limited by certain host factors, where specific *in vivo* conditions such as high sodium chloride concentrations and/or low pH can impede their performance^36^. To investigate these possibilities, we determined the inhibitory activity of 6K-F17 against planktonic *P*. *aeruginosa* isolates as a function of salt concentration ([NaCl] = 0–200 mM; PAO1 strain), and of changes in pH (range = 6–8; 007E3–2 strain) (Fig. S1). We found that the peptide’s MIC values remained unaffected by both of these sets of conditions.

**Figure 1:**
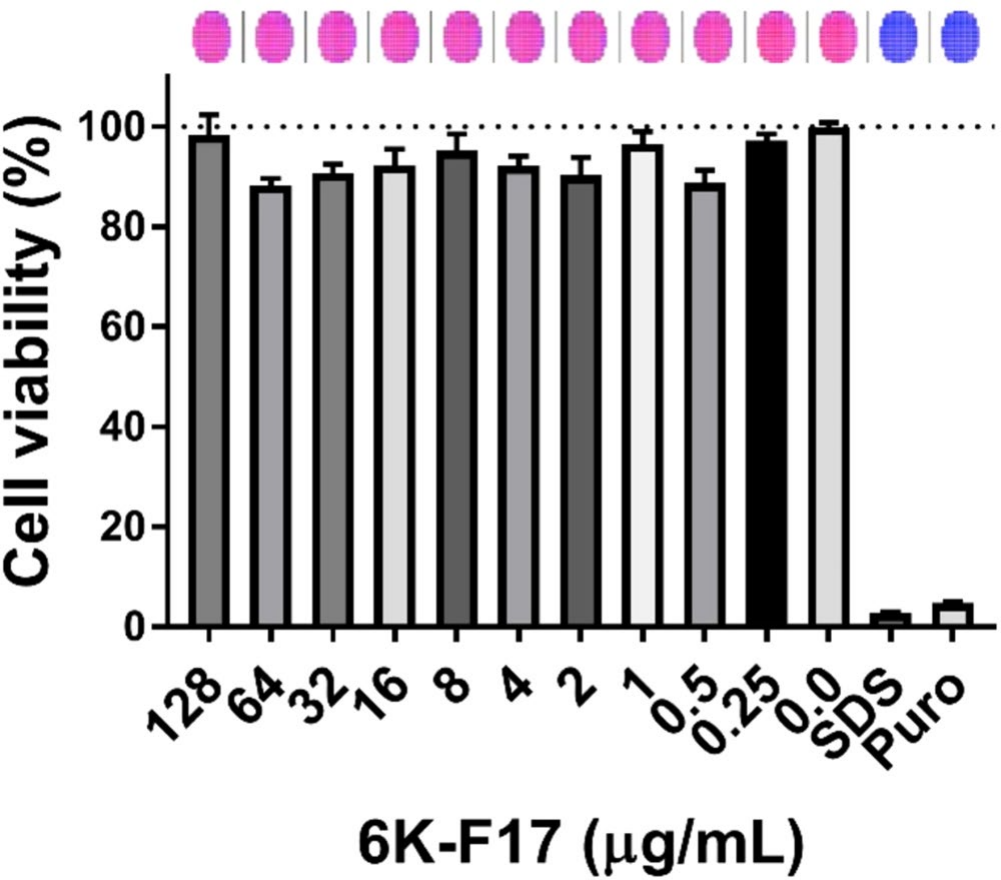
Properties of designed cationic antimicrobial peptide 6K-F17. 6K-F17 toxicity against cystic fibrosis bronchial epithelial (CFBE) cells with the F508del mutation. CFBE F508del cells were incubated overnight with increasing concentrations of 6K-F17, and toxicity was assessed by the fluorescence of Calcein-AM. The percentage of live cells was normalized to the fluorescence of cells treated with cell culture media alone (0.0 µg/mL peptide). 1% SDS detergent, and 20 µg/mL puromycin (Puro), were used as positive controls for cell death. A visual representation of the assay is shown above the graph, where live cells are depicted in pink, dead cells in blue. Values represent the average from N = 3 biological replicates; error is reported as standard error of the mean.

In addition, given the reality that much of the therapy for CF patients is administered through inhalation of antibiotics such as tobramycin, we undertook to assess a further aspect of 6K-F17 safety in a bronchial epithelial cell line derived from an individual homozygous for F508del (CFBE410^−^). Cell viability was assessed using the fluorescent live cell dye, Calcein-AM. As shown in Fig. 1, we found that 6K-F17 was not cytotoxic when tested in the CF airway cells up to 128 μg/mL.

### 6K-F17 disrupts *P*. *aeruginosa* PAO1 biofilms

**Figure 2:**
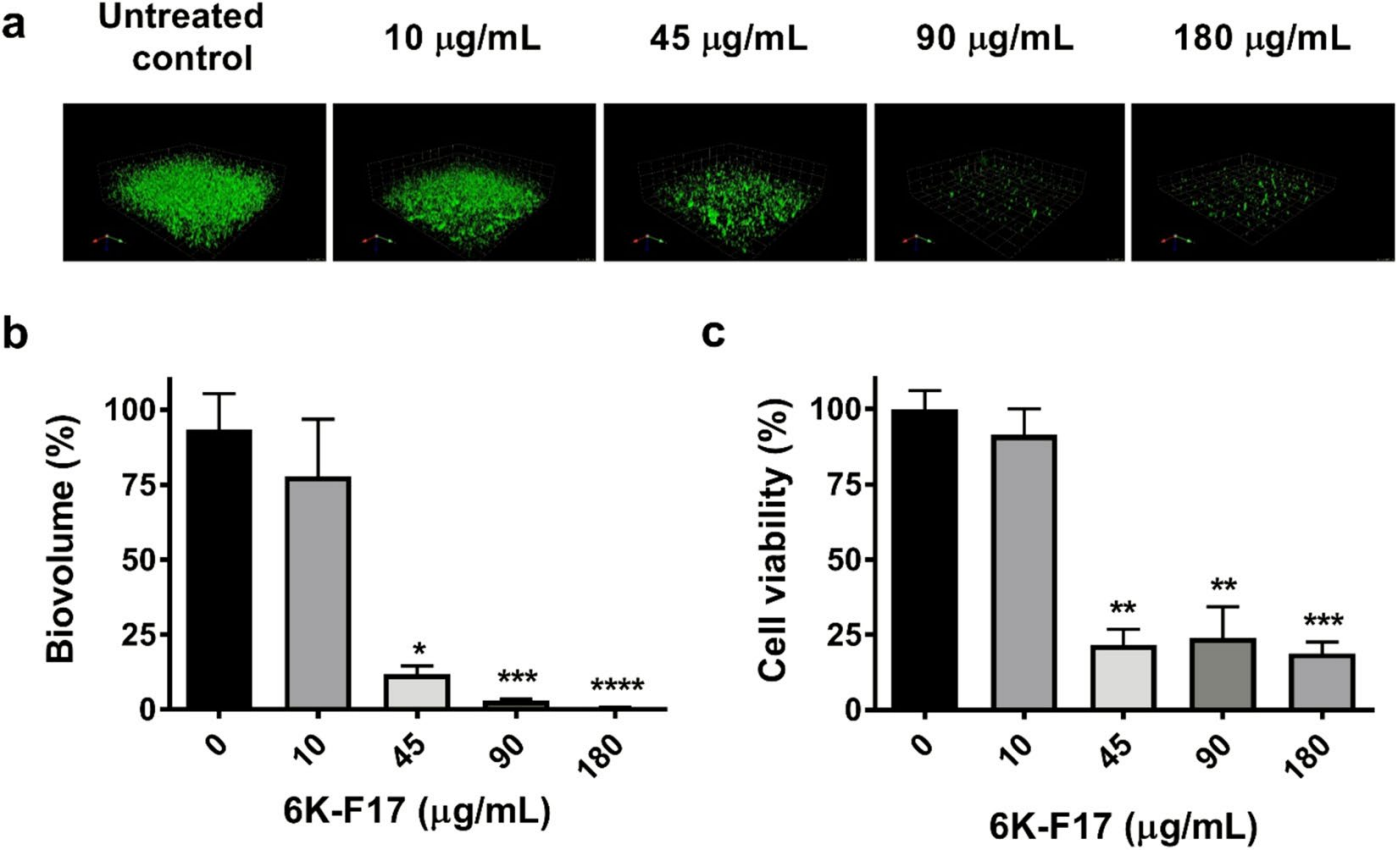
Cationic peptide 6K-F17 kills *P. aeruginosa* biofilms. (**a**) Representative images of 30-hour slide chamber *P. aeruginosa* PAO1 biofilms were grown with increasing concentrations of 6K-F17 for 16 hours prior to confocal imaging and analysis of biovolume. (**b**) Total biofilm biovolume expressed as percentage of untreated controls. The mean of n = 12 images from N = 4 biological experiments is plotted. Comparison of means between conditions was performed using a Kruskal-Wallis test with a Dunn's multiple comparison post-test. (**c**) ATP cell viability assay of PAO1 biofilms exposed to increasing concentrations of 6K-F17. PAO1 was grown in 96-well microtiter plates and ATP assays were performed as described in the methods. The mean of n = 4 experiments is plotted. Comparison of means between conditions was performed using a Mann-Whitney U test, **p < 0.01. Error is reported as standard error of the mean.

In order to determine the effect of the cationic peptide 6K-F17 on biofilm disruption, we grew the laboratory strain PAO1 in slide chambers for 30 hours followed by exposure to different concentrations of 6K-F17. As seen in Fig. 2a, increasing concentrations of 6K-F17 had the ability to disrupt 30-hour biofilms leading to reduced biovolume (Fig. 2b) and decreased number of viable cells in these biofilms (Fig. 2c). Increasing concentrations of the peptide (10–180 µg/mL) resulted in increasing disruption of biofilm (86% biovolume for 10 µg/mL to less than 3% biovolume for 180 µg/mL of the untreated control, p < 0.001) and less viable cells (20% of the viable cells in control condition). Additionally, the number of viable cells as assessed by colony forming units per mL (CFU/mL) was decreased in a dose dependent manner (Table 2).

**Table 2:** Colony forming units per mL (CFU/mL) of *P*. *aeruginosa* PAO1 exposed to various concentrations of peptide 6K-F17, tobramycin, or a combination of tobramycin and 6K-F17.

	Cells alone	6K-F17				Tobramycin				Tobramycin + 6K-F17 (10 µg/mL)			
(µg/mL)	NA	10	45	90	180	10	125	250	500	10	125	250	500
Mean log10 (CFU/mL)	12.9	10.6	4.9	NG	NG	12.3	5.0	4.0	1.2	7.3	6.5	6.0	NG
STDEV*	0.03	0.09	0.91	NA	NA	0.71	0.03	0.03	1.63	0.03	0.34	0.29	NA

NA: Non-applicable; NG: No growth; STDEV: Standard deviation. The mean of n = 2 biological replicates is shown.

*Standard deviation calculated on log transformed CFU/ml data of biological replicates.

### 6K-F17 potentiates tobramycin activity against *P*. *aeruginosa* PAO1 biofilms

**Figure 3:**
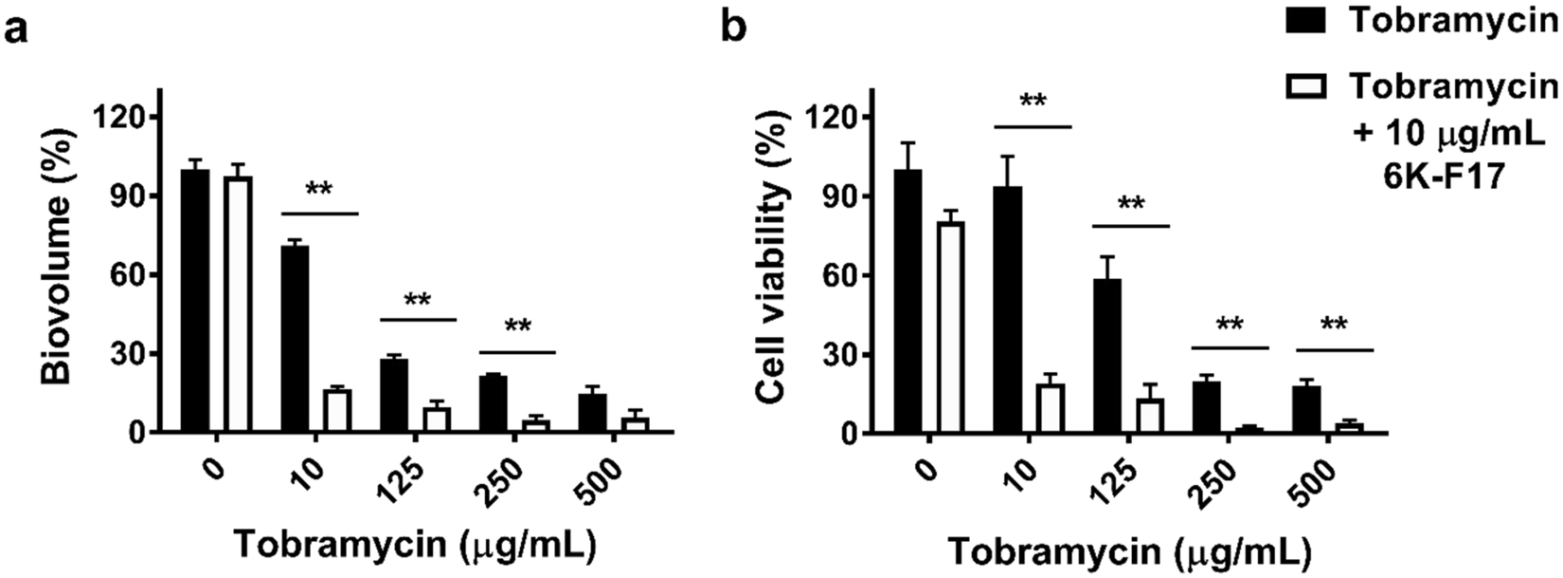
Cationic peptide 6K-F17 potentiates tobramycin activity against *P. aeruginosa* biofilms. 30-hour slide chamber *P. aeruginosa* PAO1 biofilms were grown with tobramycin alone in increasing concentrations, or with a fixed concentration (10 µg/mL) of 6K-F17 + increasing concentrations of tobramycin for 16 hours prior to confocal imaging and analysis of biovolume. (**a**) Total biofilm biovolume expressed as percentage of untreated controls. The mean of n = 12 images from N = 4 biological experiments is plotted. Comparison of means between conditions was performed using a Kruskal-Wallis test with a Dunn’s multiple comparison post-test. (**b**) ATP cell viability assay of PAO1 biofilms exposed to increasing concentrations of tobramycin alone fixed concentration (10 µg/mL) of 6K-F17 + increasing concentrations of tobramycin for 16 hours. PAO1 was grown in 96-well microtiter plates and ATP assays were performed as described in the methods. The mean of n = 4 experiments is plotted. Comparison of means between conditions was performed using a Mann-Whitney U test, **p < 0.01. Error is reported as standard error of the mean.

We next tested the ability of our peptide to enhance tobramycin activity against *P*. *aeruginosa* biofilms. To do this, we grew PAO1 in slide chambers for 30 hours. Following initial growth, biofilms were subjected to various concentrations of tobramycin with or without a fixed concentration of 6K-F17 (10 µg/mL) for 16 hours. Figure 3a demonstrates that while 10 µg/mL of 6K-F17 or low doses of tobramycin (10 µg/mL) had little impact on biofilm biovolume alone (87% and 77% of untreated control respectively), the combination of 10 µg/mL 6K-F17 and 10 µg/mL tobramycin decreased overall biofilm biovolume to less than 20% of control conditions (p < 0.001). Subsequent increases in tobramycin concentration in the presence of 10 µg/mL of 6K-F17 further decreased biofilm biovolume to 4% of the control. The addition of 6K-F17 to tobramycin also led to a reduction in the number of viable bacterial cells as assessed by the ATP assay (Fig. 3b)^37–39^ as well as CFU/mL (Table 2). There was no detectable growth in the media fraction (planktonic fraction) of tobramycin alone, or of 6K-F17 + tobramycin, suggesting that the biofilm is efficiently destroyed (*data not shown*).

**Figure 4:**
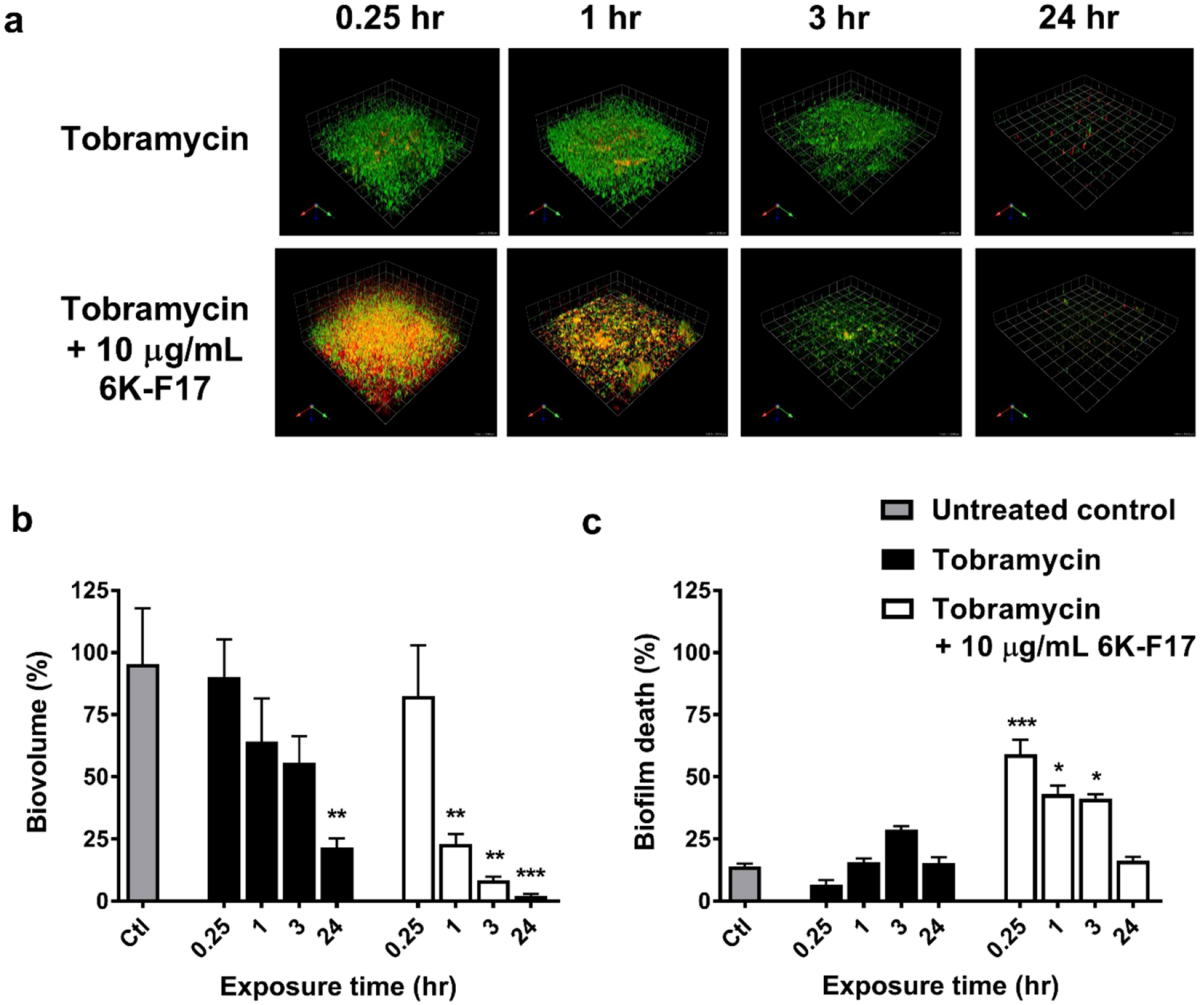
Time course for tobramycin biofilm kill compared to 6K-F17 + tobramycin. **(a)** Representative images of PAO1 biofilms exposed to 6K-F17. PAO1 was grown on slide chamber in LB media for 30 hours prior to exposure with 1000 μg/mL of tobramycin alone or 10 µg/mL of 6K-F17 + 1000 μg/mL tobramycin for increasing amounts of time. Following this, biofilms were stained with a live (green cells)/dead (red cells) cell viability kit for 1 hour prior to confocal imaging. 3D images were constructed using Volocity software. **(b)** Total biovolume expressed as a percentage of the untreated control condition. **(c)** Total biovolume that is dead/total biovolume. Note that % death decreases as total biovolume decreases. The mean of n = 12 images from N = 4 biological replicates is plotted. Comparison of means compared to untreated control was performed using a Kruskal-Wallis test with a Dunn’s multiple comparison post-test, *p < 0.05, **p < 0.01, ***p < 0.001. Error is reported as standard error of the mean.

The time required for 6K-F17 to potentiate tobramycin kill of *P*. *aeruginosa* biofilms was then investigated. Using a tobramycin concentration of 1000 µg/mL (reflecting the mean sputum concentrations achievable with inhalation therapy^40^), the addition of 6K-F17 to tobramycin resulted in significant bacterial killing within 15 minutes, while very little killing was evident with tobramycin alone (Fig. 4a). The presence of 6K-F17 increased the proportion of biofilm killed in as little as 15 minutes and resulted in significant reduction of biovolume by 3 hours (Fig. 4b,c). Taken together, these data suggest that combination of 6K-F17 with tobramycin results in biofilm killing and ultimately reduces biofilm volume more rapidly than does tobramycin alone.

### 6K-F17 potentiates the tobramycin kill of PAO1 *P*. *aeruginosa* in the presence of CF sputum

**Figure 5:**
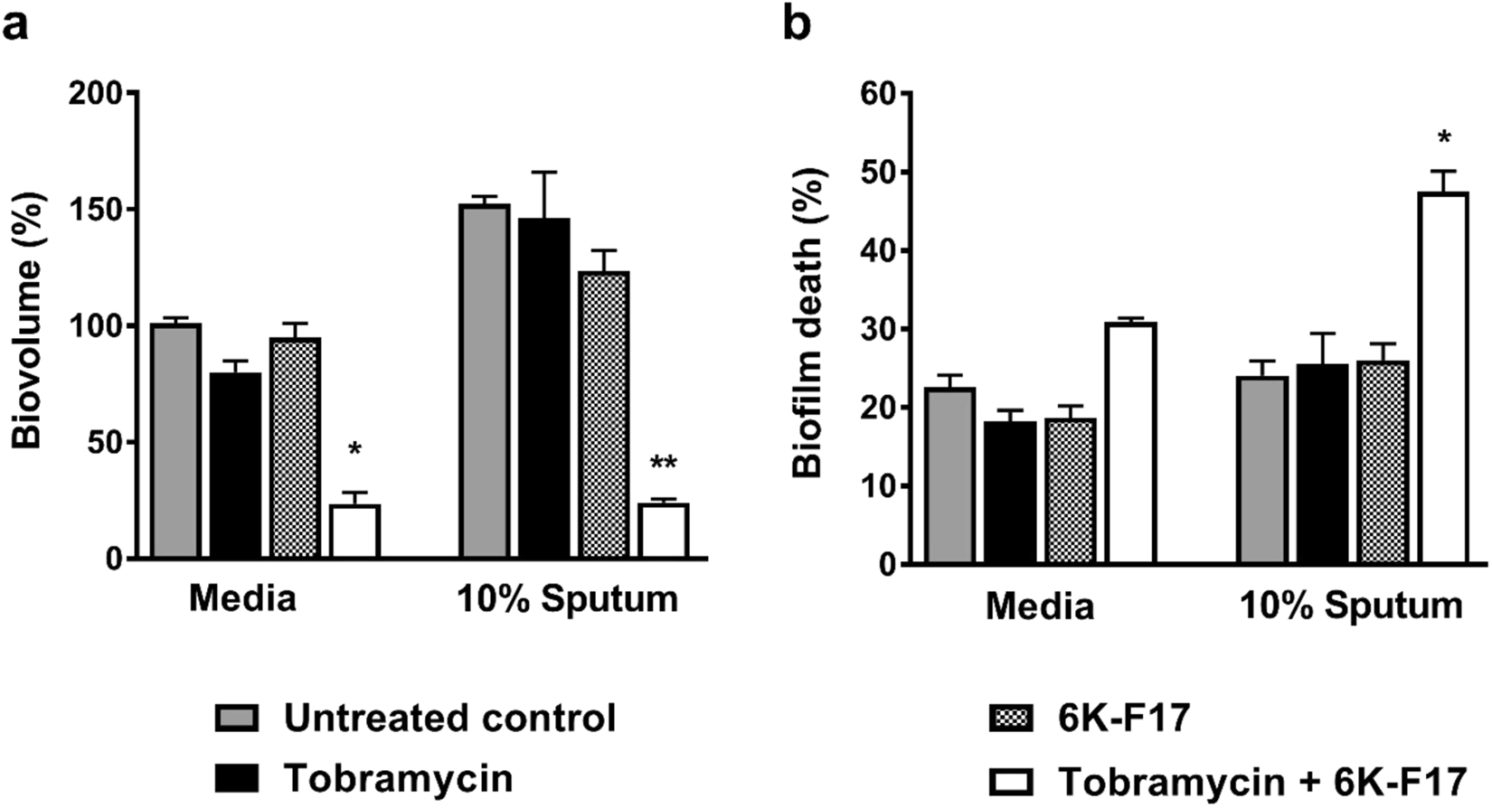
6K-F17 potentiates tobramycin activity in the presence of CF sputum. PAO1 was grown on a slide chamber in LB media alone or media with pooled sputum supernatant (10% v/v) for 48 hours prior to exposure with 100 μg/mL of tobramycin alone, or 10 μg/mL of 6K-F17 alone, or 100 μg/mL tobramycin + 10 μg/mL of 6K-F17 for 3 hours. Following this, biofilms were stained with a live/dead cell viability kit for 1 hour prior to confocal imaging. 3D images were constructed using Volocity software. (**a)** Total biovolume expressed as a percentage of the untreated control condition from the experiment. (**b)** Total biovolume that is dead/total biovolume. The mean n = 12 images from N = 4 biological replicates is shown. Comparison of means to the untreated condition was performed using a Kruskal-Wallis test with a Dunn’s multiple comparison post-test, *p < 0.05, **p < 0.01. Error is reported as standard error of the mean.

Host-derived products can often inactivate cationic microbial peptides^16^ and the addition of sputum supernatant can increase the thickness of biofilms, making them more drug resistant^41,42^. We thus tested the activity of 6K-F17 and tobramycin compared to tobramycin alone against biofilms grown in the presence of sputum supernatant collected from CF patients. As shown in Fig. 5a, neither 100 µg/mL of tobramycin alone nor 10 µg/mL of 6K-F17 alone significantly reduced the biovolume of PAO1 biofilms after 3 hours of incubation in media alone or in the presence of sputum supernatant. However, the combination of both compounds resulted in significant reduction of biofilm biovolume compared to the untreated control even in the presence of CF sputum; biovolume reduced to 23% of untreated control in media alone (p < 0.001) and reduced to 24% of untreated control in the presence of sputum (p < 0.001). Similarly, the combination of both compounds resulted in increased killing compared to untreated conditions, even in the presence of CF sputum supernatant (p < 0.05) (Fig. 5b).

### 6K-F17 disrupts biofilms from multidrug resistant *P*. *aeruginosa* clinical isolates

**Figure 6:**
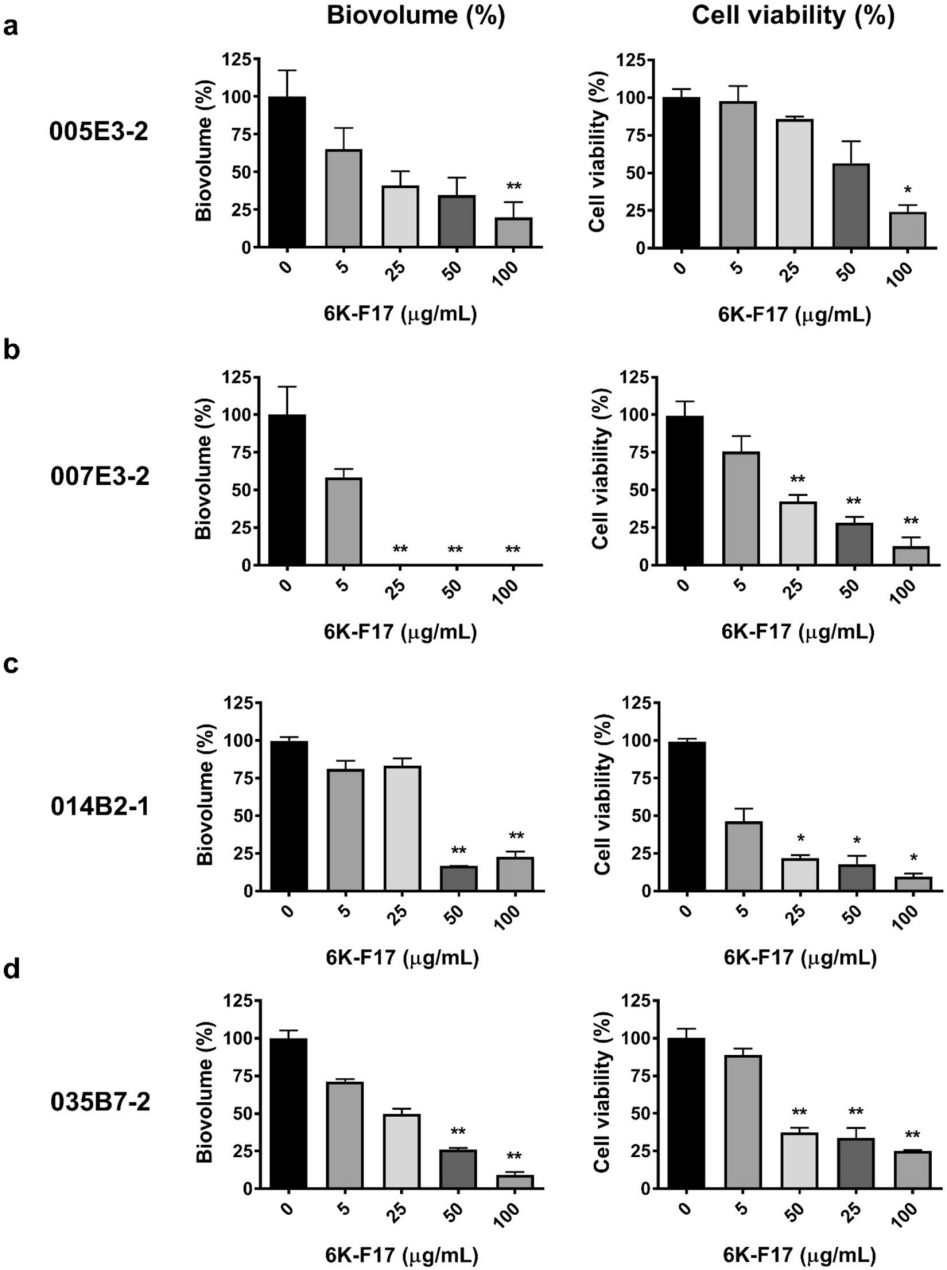
Cationic peptide 6K-F17 kills of multidrug resistant clinical isolates of *P. aeruginosa* biofilms. (**a**–**d**) Four multidrug resistant clinical *P. aeruginosa* isolates from chronically infected CF patients, were grown on a slide chamber in LB media for 30 hours prior to exposure with 6K-F17 for 16 hours prior to staining and confocal imaging. Left panels illustrate the biovolume as a percent of the untreated control. The mean of n = 12 images from N = 4 biological experiments is plotted and compared between conditions using a Kruskal-Wallis test with a Dunn’s multiple comparison post-test. Right panels illustrate the ATP bacterial viability assay (mean of n = 4 experiments). Comparison of means between the two conditions was done using a Mann-Whitney U test, **p < 0.01. Error is reported as standard error of the mean.

In addition to testing the activity against the laboratory strain PAO1, we measured the ability of 6K-F17 to disrupt biofilms of multidrug resistant CF clinical isolates of *P*. *aeruginosa*. Four clinical isolates of *P*. *aeruginosa* were collected from CF patients with chronic *P*. *aeruginosa* infection followed at the Hospital for Sick Children (Toronto, Canada). The antibiotic susceptibility results and mucoidy status of these isolates are shown in Table S1. Figure 6 illustrates the biofilm biovolume and viability after 30 hours of growth once exposed to varying concentrations of 6K-F17 for 16 hours. Figure 6b (isolate 007E3-2) and Fig. 6d (isolate 035B7-2) demonstrate significant disruption and killing of the biofilm at 6K-F17 concentrations as low as 45 µg/mL, whereas Fig. 6a (isolate 005E3-2) and Fig. 6c (014B2-1) show a more modest response to the peptide.

**Figure 7:**
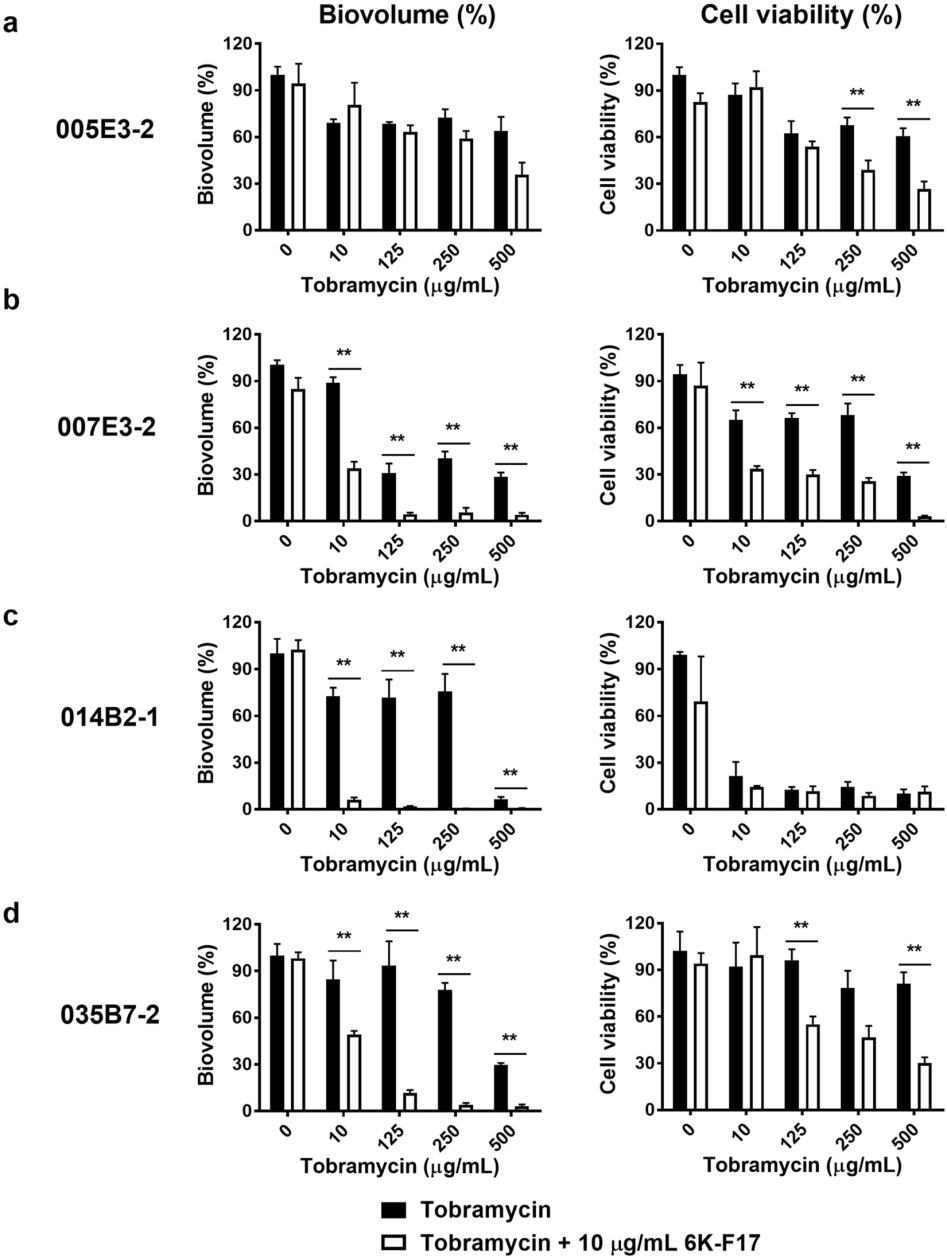
Cationic peptide 6K-F17 potentiates tobramycin kill of multidrug resistant clinical isolates of *P*. *aeruginosa* biofilms. **(a–d)** Four multidrug resistant clinical *P*. *aeruginosa* isolates from chronically infected CF patients, were grown on a slide chamber in LB media for 30 hours prior to exposure with tobramycin alone in increasing concentrations, or with a fixed concentration (10 µg/mL) of 6K-F17 + increasing concentrations of tobramycin for 16 hours prior to staining and confocal imaging. Left panels illustrate the biovolume as a percent of the untreated control. The mean of n = 12 images from N = 4 biological experiments is plotted and compared between conditions using a Kruskal-Wallis test with a Dunn’s multiple comparison post-test. Right panels illustrate the ATP bacterial viability assay (mean of n = 4 experiments). Comparison of means between the two conditions was done using a Mann-Whitney U test, **p < 0.01. Error is reported as standard error of the mean.

Finally, we examined the ability of 6K-F17 to potentiate tobramycin biofilm disruption and killing of these multidrug resistant CF clinical isolates. Figure 7 demonstrates that 6K-F17 potentiated the effect of tobramycin in reducing the biovolume and viability of clinical isolate 007E3-2 (Fig. 7b), 014B2-1 (Fig. 7c) and 035B7-2 (Fig. 7d). For isolate 005E3-2, however, the addition of 6K-F17 significantly reduced the biovolume (compared to tobramycin alone) only at tobramycin concentrations of 500 µg/mL (Fig. 7a).

## Discussion

Antimicrobial peptides have long been investigated as antimicrobial agents against a variety of different bacterial species^43–45^. Drug development has been limited primarily by safety concerns and the ability of the compounds to function *in vivo*^15^. In the present study, we demonstrated that the novel designed peptide 6K-F17 can act both as an antimicrobial at higher concentrations as well as a potentiator of a conventional antibiotic (tobramycin) at concentrations 100-fold lower than the hemolytic dose, providing a wide therapeutic window. A low dose (10 µg/mL) of 6K-F17 in combination with tobramycin killed *P*. *aeruginosa* biofilms quickly (within 15 minutes) and for a prolonged period (up to 24 hours), both desirable qualities for an antimicrobial agent. As a potentiator, 6K-F17 was able to decrease the effective required dose of tobramycin from 1,000 µg/mL to 10 µg/mL. Although clinical trials of nebulized inhaled tobramycin in CF have reported mean sputum tobramycin concentrations of 1,000 µg/mL, there is a wide range of measured levels and non-homogeneous ventilation that can lead to lower drug concentrations in certain regions of the lung^40,46–48^. Adding a potentiator such as 6K-F17 to tobramycin may thus improve the latter’s clinical efficacy. The potentiating effect of 6K-F17 may also be useful when administering tobramycin intravenously, as systemic drug administration is known to result in lower sputum concentrations compared to aerosolized treatment^46^.

We additionally showed that 6K-F17 can kill *P*. *aeruginosa* biofilms in the presence of sputum collected from CF patients, which is known to increase biofilm thickness and contains potentially inactivating compounds such as proteases, elastases, and bacterial DNA^41^. To our knowledge, this has not been previously demonstrated and suggests that 6K-F17 will be effective in the actual environment of the CF airways. Furthermore, we showed the efficacy of 6K-F17 against mature, established biofilms (48 hour growth) of multidrug resistant CF clinical isolates, to reflect a more accurate, clinical scenario^49^; Bomberger *et al*. similarly demonstrated the efficacy of their engineered CAP against clinical *P*. *aeruginosa* isolates grown as biofilms for 6 hours on airway epithelial cells, at low pH and in high salt concentrations^29^.

The molecular origin of the *de facto* synergism we observed for 6K-F17 in potentiating tobramycin activity remains a subject of current investigation. The primary mechanism of 6K-F17 antimicrobial action is believed to involve bacterial plasma membrane damage – where no protein or polysaccharide target is specifically involved^34^. In this context, it is striking that 6K-F17 nevertheless displays a range of anti-biofilm activity against the various *P*. *aeruginosa* clinical isolates studied herein. For example, 6K-F17 disrupted the biofilms of isolates of strains 007-E3-2 and 035-B7-2 more effectively than the biofilms of isolates 005-E3-2 and 014B2-1; of note, the latter two isolates were also resistant (or of intermediate resistance) to colistin, whereas the former two isolates were colistin susceptible (Table S1). Colistin is a cationic, cyclic peptide antibiotic belonging to the polymyxin family that, in principle, has a similar mechanism of action as antimicrobial peptides, by binding to bacterial cell membranes causing cell lysis and death^50^. *P*. *aeruginosa* resistance to colistin has been described through the modification by the bacteria of the lipid A component of lipopolysaccharides (LPS), the binding site of colistin^51^. Thus, the high affinity of CAPs for LPS may make them susceptible to LPS modifications (*viz*., by rendering the bacterial surface less negatively-charged) - a well-known adaptive response of *P*. *aeruginosa* to the CF lung environment - and ultimately explain the strain-strain variability in effectiveness^8,18,52^. Indeed, lipid A modification in *P*. *aeruginosa* has been shown to be associated with resistance to selected CAPs^53^. In any case, given the energy expended in modifying bacterial cell membranes, the threshold for the development of resistance against CAPs is thought to be much higher than for small molecule antibiotics.

As an additional mechanistic consideration, the varying expression level(s) of membrane-embedded efflux pumps among clinical strains is likely to be a prime factor underlying the wide range of effectiveness of both CAPs and tobramycin^54^ that we observed herein. And in a further aspect of this activity, we showed in previous *in vitro* work that 6K-F17 can form insoluble stereospecific complexes with the anionic polysaccharide alginate – a common component of the exopolysaccharide (EPS) matrix in biofilms – suggesting a ‘dual-action’ mechanism wherein 6K-F17 may disrupt not only the bacterial plasma membrane but also the biofilm EPS, thereby allowing increased access of both 6K-F17 and tobramycin (in combination experiments; Fig. 3) to the bacterial membrane^55^.

Although we sought to re-create the *in vivo* CF environment using patient sputum, our model did not include elements of the host, such as airway epithelial (AECs) and inflammatory cells. CAPs can act as immunomodulators, recruiting polymorphonuclear cells and modulating the release of pro- or anti-inflammatory cytokines, which may influence its ultimate activity^26^. Previous studies have investigated the antimicrobial effects of CAPs against *P*. *aeruginosa* biofilms grown on AECs, but these experiments are limited by the length of time one can grow a biofilm on AECs without causing cell death (typically 6 hours), resulting in more immature biofilms that are easier to kill^29^. Furthermore, the cell viability assay may not detect metabolically dormant, persister cells. It therefore remains of importance to develop adequate animal models of chronic respiratory biofilms in which to test the *in vivo* efficacy of CAPs. The spectrum of antimicrobial activity of 6K-F17 as well as its ability to potentiate other antibiotics (in addition to tobramycin) remains to be determined and is currently under investigation.

In summary, we have shown the ability of the designed CAP 6K-F17 to disrupt *P*. *aeruginosa* biofilms – both PAO1 and several multidrug resistant clinical isolates obtained from chronically infected CF patients – in a dose dependent manner. Importantly, the peptide was able to maintain its activity against *P*. *aeruginosa* biofilms in the presence of CF sputum supernatant. Low doses of 6K-F17 were able to potentiate tobramycin kill of *P*. *aeruginosa* biofilms, suggesting the potential ability to co-administer the peptide to increase the utility of tobramycin against resistant isolates. As well, 6K-F17 was able to reduce the overall biovolume of established *P*. *aeruginosa* biofilms as well as reduce the number of viable cells present. These findings portend the further development and ultimate therapeutic utility of peptide antibiotics such as 6K-F17 in the treatment of respiratory infections.

## Methods

### Bacterial isolates and sputum samples

PAO1 was used as a general laboratory strain for these experiments^56^. Clinical isolates of *P*. *aeruginosa* [Research Ethics Board (REB) #1000019444] and sputum samples (REB #100011132) were obtained with informed consent from CF patients with chronic infection followed at the Hospital for Sick Children (Toronto, Canada). Consent was obtained from a parent or legal guardian if not of age. All methods were performed in accordance with the relevant guidelines and regulations for research involving human subjects at the Hospital for Sick Children. Antimicrobial susceptibility testing was performed as per the Clinical Laboratory Standards Institute (CLSI)^57^.

### Peptide synthesis and purification

6K-F17 was synthesized by the continuous flow Fmoc solid-phase method on a Protein Technologies PS3 peptide synthesizer using the standard cycle^58^. (Fmoc-aminomethyl-3,5-dimethoxyphenoxy)-valeric acid-polyethylene glycol-polystyrene resin was used to produce an amidated C-terminus. 2-(7-Aza-1*H*-benzotriazol-1-yl)-1,1,3,3-tetramethyluronium hexafluorophosphate and *N*,*N*-diisopropylethylamine were used as the activation pair. Deprotection and peptide cleavage was performed in a mixture of 88% TFA, 5% phenol, 5% water, and 2% triisopropylsilane for 2 h in the dark at room temperature. Crude peptide was purified on a reverse-phase C4 preparative HPLC column using a linear gradient of acetonitrile in 0.1% TFA. Purity and identity of the peptide were confirmed using MALDI-MS. Peptide concentration was determined using amino acid analysis.

### MIC values of 6K-F17 and tobramycin against *P*. *aeruginosa* clinical isolates

The antibacterial activity was tested in sterile 96-well plates in a final volume of 100 μL by following standard microtiter dilution protocols in Mueller-Hinton Broth/not cation-adjusted (MHB). *P*. *aeruginosa* PAO1 or clinical isolate cells were grown in MHB at 37 °C for overnight and were diluted in the same medium to a final concentration of 5 × 10^5^ colony forming units (CFU/mL) as determined by optical density at 600 nm. Aliquots of 10 μL of serial two-fold dilutions in water of the lyophilized peptide (or tobramycin) were added to microtiter plates followed by 90 μL of bacterial suspension. Peptide (tobramycin) antibacterial activity was expressed as the MIC – the lowest concentrations that resulted in 100% prevention of bacterial growth after 24 hours incubation at 37 °C (Table 1). Optical density was measured at OD_600_ using a microplate autoreader Spectrophotometer. Positive controls contained no peptide and showed visible turbidity after 24 hours incubation at 37 °C. All assays were carried out in triplicate; repeated tests were within one dilution (standard error of the test).

### Peptide toxicity assay in human airway cells

Peptide toxicity to human cystic fibrosis bronchial epithelial cells (CFBE) containing the mutation F508del was tested using a Calcein-AM based fluorescence assay^59^. CFBE410^−^ F508del cells (kind gift from the Dieter Gruenert laboratory, California Pacific Medical Center Research Institute, San Francisco, CA) were grown on 96-well black clear bottom plates, as previously described for this immortalized cell line^60^. Media used to culture the cells were EMEM (Wisent Bio Products, Saint-Jean-Baptiste, Canada) containing 10% Fetal Bovine Serum (Wisent Bio Products, Saint-Jean-Baptiste, Canada), 300 µg/mL Hygromycin, and 1% penicillin/streptomycin. These cells were grown to confluence and then differentiated for 5 days. The cells were then treated with 6K-F17 (128 µg/mL to 0.0 µg/mL), or puromycin (20 µg/ml). On the day of the experiment, cells were washed with PBS, and then loaded with live cell marker Calcein-AM (10 µM) (Thermo Fischer Scientific, MA, USA) along with either the 6K-F17 peptide, with puromycin, or with 1% (v/v) SDS, for 30 minutes. Plates were then read using a fluorescence multi-plate reader (Molecular Devices i3x), at excitation maximum of 490 nm and emission maximum of 515 nm. Multiple points were read in the same well to account for heterogeneity across the well. The bar graph represents average fluorescence across the well (n = 137 technical replicates per well of a 96 well plate, n = 3 wells per condition, n = 3 biological replicates from independent plates).

### Colony forming units from biofilms

*Pseudomonas aeruginosa* was subcultured 1/100 from an overnight culture and grown to an OD_600_ of 0.06 in cation adjusted Muller-Hinton broth (CAMHB) at 37 °C. 100 μL of this broth was then added to wells of a 96-well polystyrene microtiter plate (Fisher, non-tissue cultured) and grown for 16 hours without shaking at 37 °C. Following this growth, media was removed, and wells were washed gently with PBS 2x prior to addition of peptide or tobramycin in CAMHB. Biofilms were grown in presence of antibiotics for 24 hours at 37 °C. After growth period, media was removed, and wells were washed twice with PBS. 100 μL of fresh PBS was then added to the wells and the plate was vortexed for 5 min. Media from wells were then serially diluted and plated on blood agar plates. Plates were grown for 24–48 hours at 37 °C and CFU were counted. CFU counts were log transformed as previously described, with the mean and standard deviations reported^61^.

### Bacterial viability assay

Cell viability of biofilms was assessed using an adapted method of the ATP assay for biofilms^62^. Briefly, *P*. *aeruginosa* isolates were grown to stationary phase at 37 °C with shaking at 200 RPM in cation-adjusted Muller-Hinton broth (CAMHB-Sigma-Aldrich.). This culture was then diluted to an OD_600_ of 0.05 and grown to an OD of 0.1 (early exponential phase) prior to plating. 100 µL of this culture were added to a white Grenier LUMITRAC medium binding plate (Sigma-Aldrich, city, country) in triplicate and incubated for 24 hours at 37 °C without shaking. After 24 hours, media were removed, and cells were gently washed 2X with fresh media. 100 µL of fresh CAMHB was added back to the wells along with 100 µL of antibiotic, cationic peptide 6K-F17, or antibiotic plus peptide, for a final volume of 200 µL and mixed gently for 5 minutes on an orbital shaker. The plate was then incubated for 16 hours at 37 °C. Following these procedures, 100 µL from each well was removed and placed into an empty well of a 96-well plate to monitor ATP in the planktonic or detached fraction of the wells. 100 µL of the Bac-titer glo ATP cell viability solution (Promega, Madison, WI) was added to each well. Plates were gently mixed on an orbital shaker for 10 minutes prior to luminescence reading as per manufacturer’s suggested protocol.

### Biofilm growth in chamber slides

*P*. *aeruginosa* was grown in chamber slides as previously described^63^. Briefly, clinical isolates of *P*. *aeruginosa* were grown overnight in 3 mL of lysogeny broth (Lennox formulation-LB) with shaking overnight. 40 μL of overnight culture was diluted into 4 mL of LB. This was diluted 1/10 and 220 µL was used to seed the wells of an 8-chambered cover-glass slide (Nunc Lab-tek II, VWR, Mississauga, Ontario, Canada). After 6 hours of attachment, media were removed and replaced with fresh media. Biofilms were allowed to grow for a further 24–48 hours, replacing media every 12 hours until the conclusion of the experiment. Pre-formed biofilms in chamber slides were also exposed to varying concentrations of cationic peptide, tobramycin (Sigma-Aldrich, Oakville, Ontario, Canada) or a combination of both as described. 24-hour biofilms were grown as described above prior to addition of various antibiotics for the indicated time periods.

### Confocal microscopy

Prior to confocal microscopy, biofilms were stained using the Filmtracer Live/Dead biofilm viability kit (Life Technologies, Burlington, ON, Canada). Medium was gently removed from the chambers, and 200 μl total of the Live/Dead stain was added to the chambers. After 45 min of incubation, the stain was removed, and fresh medium was placed in the wells. Confocal images were then acquired using a Quorum WaveFX spinning disk confocal system (Quorum Technologies Inc., Guelph, Canada). All images were acquired using a 25X water objective (total magnification, X250) on a Zeiss AxioVert 200 M Microscope. Spectral borealis lasers (green, 491 nm; red, 561 nm) were used for excitation. Emission filter sets of 515/40 and 624/40 were used to visualize the SYTO9 and propidium iodide stains, respectively. Images in the z-stack were obtained at a distance of 0.8 μm to obtain the depth of the biofilm. For each experiment, each isolate tested was performed in duplicate technical replicate (two chambers per condition) with three biological replicates (three separate experiments). Volocity software (PerkinElmer, Guelph, Canada) was used for acquisition and analysis of images.

### Sputum processing

Fresh sputum samples were collected with informed consent from CF patients at the Hospital for Sick Children (Toronto, Canada) according to the experimental protocols and guidelines for research involving human subjects as approved by the Hospital for Sick Children [Research Ethics Board (REB) #100011132]. Samples were stored on ice, and PBS was added to samples within 1 hour of collection at 3 times the volume of the sputum. Samples were vortexed for 5 min and 1 mL aliquots were placed into 1.5 mL microcentrifuge tubes and spun down at 14,000 g for 10 min at 4 °C. Supernatant was removed gently and filtered sterilized through a 20 µm low-binding filter. The sample was plated on blood agar for 48 hours at 37° to ensure no bacterial growth. Sputum samples were pooled from 5 patients.

### Statistical analysis

Continuous data were compared within groups, as performed using the Kruskal-Wallis test with a Dunn’s multiple comparison post-test. For comparisons between tobramycin alone and tobramycin + peptide, means where compared with multiple t-tests with the Holm-Sidak method for multiple comparisons. A *P-*value of < 0.05 was considered significant. All analyses were done using GraphPad Prism version 6.01.

## Electronic supplementary material

Supplementary information

## Data Availability

All data generated or analyzed during this study are included in this published article (and its Supplementary Information files).
