# Supplementary material for: Activity of a novel antimicrobial peptide against Pseudomonas aeruginosa biofilms
Source: Sci Rep. 2018 Oct 3;8:14728. doi: 10.1038/s41598-018-33016-7 (PMC6170476; doi:10.1038/s41598-018-33016-7)
Supplement: Supplementary file 1 — Supplementary information [file 41598_2018_33016_MOESM1_ESM.pdf]

Supplementary Information

Activity of a novel antimicrobial peptide against *Pseudomonas aeruginosa* biofilms

Trevor Beaudoin[1], Tracy A. Stone[2,3], Miroslawa Glibowicka[2], Christina Adams[1], Yvonne Yau[5,6], Saumel Ahmadi[2,4], Christine E. Bear[2,3,4], Hartmut Grasemann[1,6,7], Valerie Waters[1,6,8], and Charles M. Deber [2,3]

[1]Division of Translational Medicine, Research Institute, Hospital for Sick Children, Toronto, Canada.

[2]Division of Molecular Medicine, Research Institute, Hospital for Sick Children, Toronto, Canada.

[3]Department of Biochemistry, University of Toronto, Toronto, Ontario, Canada.

[4]Department of Physiology, University of Toronto, Toronto, Ontario, Canada.

[5]Division of Microbiology, Department of Pediatric Laboratory Medicine, Hospital for Sick Children, Toronto, Canada.

[6]Department of Laboratory Medicine and Pathobiology, University of Toronto, Toronto, Ontario, Canada.

[7]Division of Respiratory Medicine, Department of Pediatrics, Hospital for Sick Children, Toronto, Canada.

[8]Division of Infectious Diseases, Department of Pediatrics, The Hospital for Sick Children, University of Toronto, 555 University Avenue, Toronto, Canada M5G 1X8.

Corresponding author:
Dr. Charles M. Deber
Research Institute, The Hospital for Sick Children
Division of Molecular Medicine
PGCRL, Room 20.9712
686 Bay Street
Toronto, ON M5G 0A4
deber@sickkids.ca

Methods

Antimicrobial susceptibility of clinical isolates of *P. aeruginosa*

Conventional susceptibility testing was performed by broth microdilution using Sensititre GN2F panels (Trek Diagnostic Systems, Oakwood Village, Ohio, USA) according to manufacturer's instructions except for chloramphenicol and colistin. For these two antibiotics, susceptibility was performed by disk diffusion according to CLSI guidelines [Ref.: Clinical Laboratory Standards Institute. *Performance standards for antimicrobial disk susceptibility testing; approved standards.* M02-A11. Wayne, PA., 2012]. Susceptibility panels and plates were incubated aerobically at 37° C for 24 hours. The values reported in Table S1 were obtained by these protocols.

Minimum inhibitory concentration (MIC) of 6K-F17 as a function of sodium chloride concentration

The effect of salt concentration on the antimicrobial activity of the 6K-F17 peptide was tested by determining the MICs of the peptide at various salt concentrations ranging from 0-200 mM. Experiments were performed under aerobic conditions in sterile 96-well microtiter plates (Costar) in a final volume of 100 μL by following standard microtiter dilution protocols in MHB. *P. aeruginosa* PAO1 strain was maintained as a glycerol stock at -80°C. Bacterial cells were grown in MHB at 37°C for overnight and were

diluted in the same medium to a final concentration of 5 x 10^5 colony forming units (CFU/mL) as determined by optical density at 600 nm. Twofold serial dilutions of peptide, with concentration ranging from 64 μg/ml to 1 μg/mL were diluted in water. Bacteria (90 μl) and peptide (10 μl) were added to the wells of sterile 96-well polypropylene microtiter plates. Positive controls contained no peptide and demonstrated visible turbidity. Plates were incubated at 37°C overnight and read at 600 nm in a microplate reader (Molecular Devices). Peptide antibacterial activity was expressed as the MIC – the lowest peptide concentration that resulted in 100 % prevention of microbial growth. All assays were carried out in triplicate. The results are displayed in Supplemental Fig. S1, left panel.

MIC of 6K-F17 as a function of pH

The antimicrobial activity of 6K-F17 was tested at points between pH 6 and pH 8 under aerobic conditions in sterile 96-well microtiter plates (Costar) in a final volume of 100 μL by following standard microtiter dilution protocols in MHB. The pH of the media was adjusted with 6N HCl or NaOH. *P. aeruginosa* clinical strain HSC 007E3-2 was maintained as a glycerol stock at -80°C. Bacterial cells were grown in MHB at 37°C for overnight and were diluted in the same medium to a final concentration of 5 x 10^5 colony forming units (CFU/mL) as determined by optical density at 600 nm. Twofold serial dilutions of peptide, with concentrations ranging from 128 μg/ml to 1 μg/mL were diluted in water. Bacteria (90 μl) and peptide (10 μl) were added to the wells of sterile

96-well polypropylene microtiter plates. Positive controls contained no peptide and demonstrated visible turbidity. Plates were incubated at 37°C overnight and read at 600 nm in a microplate reader (Molecular Devices). Peptide antibacterial activity was expressed as MIC. All assays were carried out in duplicate. The results are displayed in Fig. S1, right panel.

Table S1. Antimicrobial susceptibility of clinical isolates of *P. aeruginosa*[a]

	005E3-2	007E3-2	014B2-1	035B7-1
**Amikacin**	I	R	S	R
**Aztreonam**	R	R	R	R
**Cefepime**	R	R	I	S
**Ceftazidime**	R	R	I	R
**Ciprofloxacin**	I	R	R	R
**Gentamicin**	R	R	R	R
**Meropenem**	R	R	R	S
**Piperacillin**	R	R	R	R
**Piperacillin-tazobactam**	R	R	R	R
**Tobramycin**	R	R	R	R
**Colistin**	R	S	I	S
**Chloramphenicol**	R	R	R	R
**Mucoid Status**	Mucoid	Non-mucoid	Non-mucoid	Mucoid

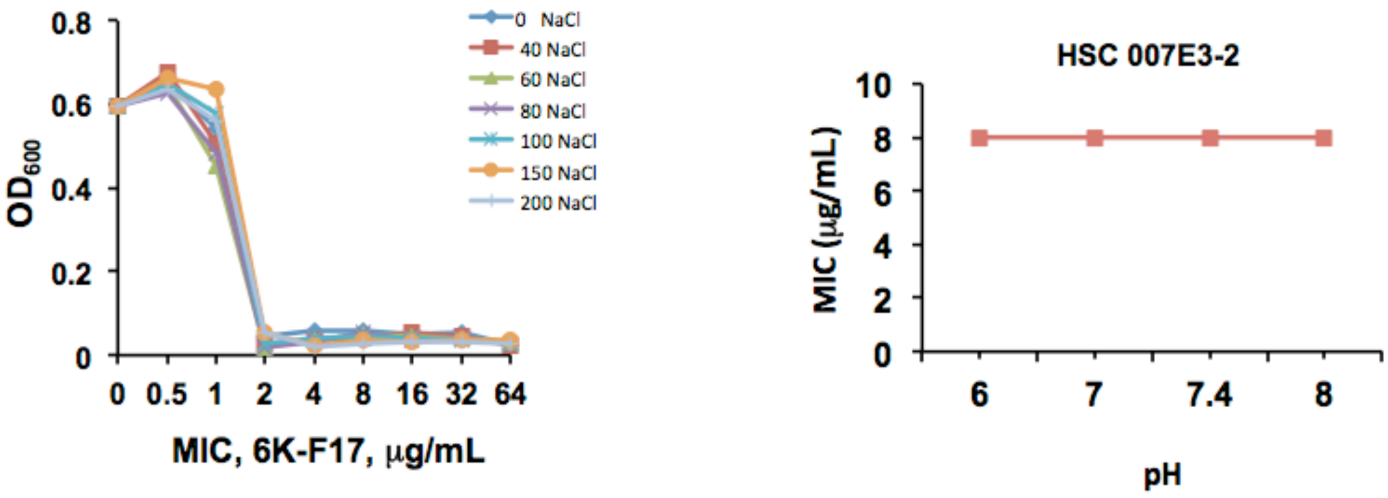

Figure S1. Dependence of 6K-F17 MIC as a function of sodium chloride concentration or pH. *Left panel*: *P. aeruginosa* PAO1 strain was incubated for 24 hours in LB containing various concentrations of sodium chloride (mM), as indicated in the diagram, with various concentrations of 6K-F17, after which the OD was recorded. *Right panel: P. aeruginosa* MDR isolate 007E3-2 was incubated for 24 hours in LB in the presence of 6K-F17 at values between pH 6 and pH 8. MICs are reported at the peptide concentration where zero bacterial growth was observed. All experiments in both panels were performed in triplicate; MIC values typically fell within one dilution. See Supplemental Methods for experimental details.
